# *xrayutilities*: a versatile tool for reciprocal space conversion of scattering data recorded with linear and area detectors

**DOI:** 10.1107/S0021889813017214

**Published:** 2013-07-18

**Authors:** Dominik Kriegner, Eugen Wintersberger, Julian Stangl

**Affiliations:** a Institute of Semiconductor and Solid State Physics, Johannes Kepler University, Altenbergerstrasse 69, A-4040 Linz, Austria; b DESY, Notkestrasse 85, D-22607 Hamburg, Germany

**Keywords:** X-ray scattering, data analysis, reciprocal space, computer programs

## Abstract

General algorithms to convert scattering data of linear and area detectors recorded in various scattering geometries to reciprocal space coordinates are presented. These algorithms work for any goniometer configuration including popular four-circle, six-circle and kappa goniometers. The use of commonly employed approximations is avoided and therefore the algorithms work also for large detectors at small sample–detector distances. A recipe for determining the necessary detector parameters including mostly ignored misalignments is given. The algorithms are implemented in a freely available open-source package.

## Abstract 2

Algorithms for the reciprocal space conversion of linear and area detectors are implemented in an open-source Python package.

## 1. Introduction

Elastic X-ray scattering is a very widely applied technique to study the structure of materials ranging from single crystals, powders and other forms of hard condensed matter to biological tissues and organic molecules. Crystalline as well as noncrystalline matter such as liquids and amorphous materials can be studied by techniques like X-ray diffraction and reflectometry. A variety of approaches exist to analyze the scattering data. Some quantities can be directly determined from the measurements [*e.g.* lattice parameters (Bond, 1960 ▶; Fewster, 1999 ▶), layer thicknesses (Warren, 1969 ▶; Pietsch *et al.*, 2004 ▶)]. Other types of analysis involve simulation of the scattering signal to determine strain and material composition (Stangl *et al.*, 2004 ▶; Wintersberger *et al.*, 2010 ▶), or the model-free determination of real space structure using phase retrieval algorithms (Fienup, 1982 ▶; Miao *et al.*, 1998 ▶; Diaz *et al.*, 2009 ▶; Minkevich *et al.*, 2011 ▶). All of those approaches have in common that the analysis is most of the time performed in reciprocal space and hence requires a conversion of experimentally measured data into reciprocal space. While the particular analysis steps differ for different experiments, the conversion itself is a common step, which needs to be performed for a lot of different techniques. This has been treated very well for the case of point detectors (Busing & Levy, 1967 ▶; Lohmeier & Vlieg, 1993 ▶; You, 1999 ▶; Bunk & Nielsen, 2004 ▶). For one-dimensional and two-dimensional detectors, which are used more and more frequently, several issues related to the detector geometry and detector alignment complicate a correct conversion.

For some applications like protein crystallography or powder diffraction, experimental and analysis schemes are standardized. Examples are software packages to extract peak positions and intensities in protein crystallography (Leslie, 2006 ▶) and powder diffraction (Lutterotti *et al.*, 1999 ▶; Rodriguez-Carvajal, 2001 ▶; Hammersley, 2013 ▶), or the PDB Protein Data Bank (http://www.pdb.org/) format and the CIF crystallographic information file format established by the International Union of Crystallography for exchange of structure files and experimental data.

For most other cases, only very specialized solutions to particular experiments exist, each containing solutions for some aspects important for the respective case; for example, the experimental control software *spec* (Certified Scientific Software, 2013 ▶) used at various synchrotron sources is able to perform reciprocal space conversion for several goniometer geometries but only for point detectors. Commercial software supplied with several diffractometers is optimized for certain geometry/detector combinations.

A particular problem of one- and two-dimensional detectors is misalignment with respect to the ideal case: at zero detector angle, the line or plane of the detector should ideally be perpendicular to the incident X-ray beam. In practice, this will not be the case, even if deviations will usually be very small. For two-dimensional detectors, in addition a rotation of the detector around the incident beam direction can occur. In most cases, these misalignments will be unintentional and rather small; they are difficult to measure and hence often neglected. However, considering these effects is actually important to obtain correct and accurate reciprocal space coordinates.

We present a generally applicable algorithm for the conversion of experimental data recorded with point, linear and area detectors, and for any diffractometer with an arbitrary number and sequence of axes. For this purpose, we generalize the algorithms presented in several papers (Busing & Levy, 1967 ▶; Lohmeier & Vlieg, 1993 ▶; You, 1999 ▶; Bunk & Nielsen, 2004 ▶) to arbitrary goniometer geometries without approximations: we consider the fact that most one- and two-dimensional detectors are flat and hence the relation between channel number and scattering angle is nonlinear. Furthermore, we provide recipes to determine the necessary detector parameters from a set of simple scans around the primary beam. These scans also enable the user to determine the above-mentioned and several other misalignment parameters.

To keep our solution as general as possible, we have implemented it within a freely available software package, *xrayutilities* (Kriegner & Wintersberger, 2013 ▶). This generalized algorithm is also particularly useful for automatized tool chains as planned by several synchrotron facilities right now (*Passerelle*, 2013 ▶). In addition to the reciprocal space conversion described in this article, *xrayutilities* includes routines to read various data formats and methods to calculate experimental parameters from material properties. More information on those parts can be found on the *xrayutilities* web site (Kriegner & Wintersberger, 2013 ▶). This article focuses on the reciprocal space conversion part. After the introduction of the applied algorithms following the approach of You (1999 ▶), we show the extension for linear and area detectors and explain the use of our algorithms and how necessary parameters can be determined. We demonstrate the application of our approach for a few selected examples of both laboratory diffractometers and synchrotron beamlines. In an appendix we discuss one complete example, including the particular entries into the *xrayutilities* package required to define the diffractometer geometry, correctly initialize a two-dimensional detector setup, and convert a two-dimensional detector frame into reciprocal space.

## 2. Angular to reciprocal space conversion

Conversion of angular coordinates to reciprocal space can be tedious since one needs special equations for every diffractometer/detector geometry. For several diffraction geometries explicit formulas are given, for example, by Pietsch *et al.* (2004 ▶). However the conversion can be performed in a general way as long as the information about the goniometer geometry is available together with the experimental angles. The algorithm presented below therefore needs not just the experimental angles as input parameters but also a description of the goniometer. To work for arbitrary goniometers the physical order of the cradles, *i.e.* how they are mounted on each other, and the orientation of the rotation axes are needed. An example is given below. To unambiguously specify the rotation axes of the goniometer circles, a reference coordinate system is fixed to the laboratory frame. It is useful to choose this coordinate system in a way that the primary X-ray beam propagates along one of the coordinate axes.

The reciprocal space coordinates we want to know for each measured point are coordinates of the scattering vector 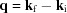, where 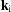 is the wavevector of the incident X-ray beam and 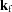 the wavevector of the scattered beam towards a particular detector (pixel) position. The coordinates of 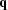 we are interested in are those in a coordinate system fixed to the sample under investigation. The coordinates of 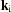 in the laboratory system are fixed by our choice of the coordinate system. The coordinates of 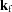 are given by the angle positions of the detector arm. To describe 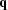 in the sample coordinate system, we also need all goniometer angles changing the sample orientation.

**Figure 1:**
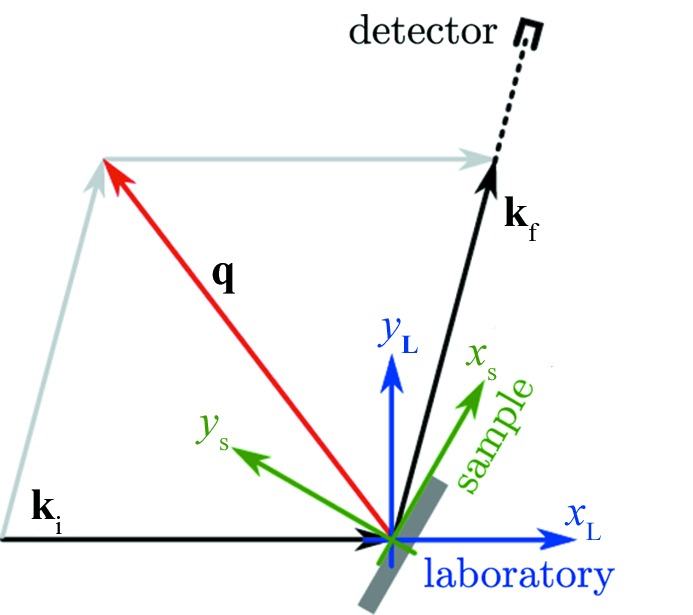
Two-dimensional sketch of the scattering process and the coordinate systems attached to the laboratory (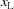,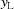) and sample (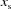, 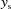). Shown are the incidence and exit wavevectors 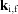 as well as the scattering vector 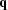. The laboratory coordinate system is chosen to have the 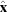 direction coinciding with the primary beam direction.

A minimal two-dimensional example illustrating the definition of our laboratory coordinate system (blue) is shown in Fig. 1 ▶. The coordinate system attached to the sample is indicated in green in Fig. 1 ▶. So what we have to deal with are coordinate transformations between the different involved coordinate systems. Those coordinate transformations can be written as matrix equations. We will reproduce some of the essential equations of earlier papers (Busing & Levy, 1967 ▶; You, 1999 ▶) so that the reader can follow the further generalization for one- and two-dimensional detectors. We consider just a point detector for the moment; the generalization for one- or two-dimensional detectors is shown in §[Sec sec3]3 below.

For the following derivations we assume that the primary beam is perfectly aligned with respect to the center of rotation of the goniometer and that the different goniometer axes are homocentric. Furthermore the sample needs to be positioned in the center of rotation of the goniometer. Deviations from these conditions will lead to inaccuracies in the measured scattering and diffraction angles and therefore introduce errors in the reciprocal space conversion described below. For further discussion on high-accuracy measurement of diffraction angles the reader should refer to Fewster (1999 ▶) and references therein.

The sample coordinate system we have been talking about is actually the coordinate system attached to the innermost goniometer circle (because this is what the goniometer angles describe). Of course, the sample can in addition have a certain orientation with respect to this circle. This is most evident in the case of a crystalline sample, where the directions of the reciprocal space of the crystal have a certain orientation with respect to the sample holder. This and the coordinates of the scattering vector in the reciprocal space of the crystal are described below by the orientation matrix 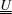 and the orthonormalization matrix 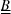, respectively. In the following, we describe this ‘most complicated’ case of a single-crystalline sample. For amorphous or powder samples *etc.*, 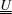 and/or 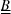 can be replaced by identity matrices.

In the crystal lattice a scattering vector might be described by the column vector
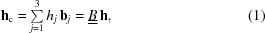
with 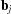 the reciprocal lattice vectors, the matrix 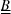 being formed from those vectors, and 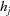 generally referred to as *h*,* k*, *l*. In the case where *h*, *k*, *l* are integer values they are called Miller indices. An explicit representation of 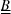 is for example given by Busing & Levy (1967 ▶) and Helliwell (2006 ▶).

To connect the scattering vector in the crystal with the scattering vector in the coordinate system attached to the sample goniometer the orientation matrix 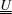 is introduced:
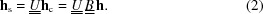
This scattering vector converted to the laboratory frame is equal to 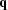. The conversion of the scattering vector is achieved by another coordinate transformation (described by matrix 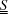), which solely depends on the sample orientation and therefore the goniometer angles that move the sample:
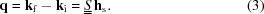
In the case of integer *h*, *k*, *l*, the former equation basically is the Laue equation.

Also any detector rotation can be expressed as a coordinate transformation described by 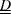, and therefore the exit wavevector is given by
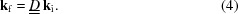



Combining equations (2)–(4) [Disp-formula fd4] one obtains the scattering vector in the crystal coordinate system: 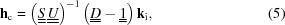
with the identity matrix 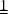. Note that all of the used coordinate transformations are transformations between two orthogonal coordinate systems and therefore yield orthogonal matrices, which are invertible. The matrix 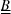, which is formed from the reciprocal space vectors 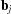 which are linearly independent, is also invertible. Since the above conversion involves a matrix inversion this is important for the algorithm to work.

The rotation matrices 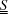 and 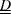 can be deduced from the description of the goniometer by multiplying the rotation matrices from each circle starting with the outermost circle: 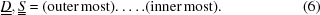



For that purpose the goniometer rotation axis needs to be defined in the laboratory coordinate system for the case when all circles are set to zero. It is therefore useful to choose the coordinate system in a way that allows this description to be as simple as possible. Keep in mind that later also the detector directions need to be determined in this coordinate system. In addition to the direction of the rotation axis, the rotation sense needs to be described. We use the mathematical definition of rotation sense. For most goniometers (except for special geometries like the κ goniometer, treated separately below) the rotation axes point along primitive directions. When looking at the rotation from the positive side of the corresponding direction, clockwise rotation is left handed, *i.e.* negative, and anticlockwise rotation is right handed (positive). For example, we will call a clockwise/left-handed/negative rotation of angle α around the *x* axis an ‘x-’ rotation, described by the following rotation matrix: 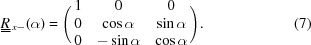



### 2.1. Example of a goniometer definition

**Figure 2:**
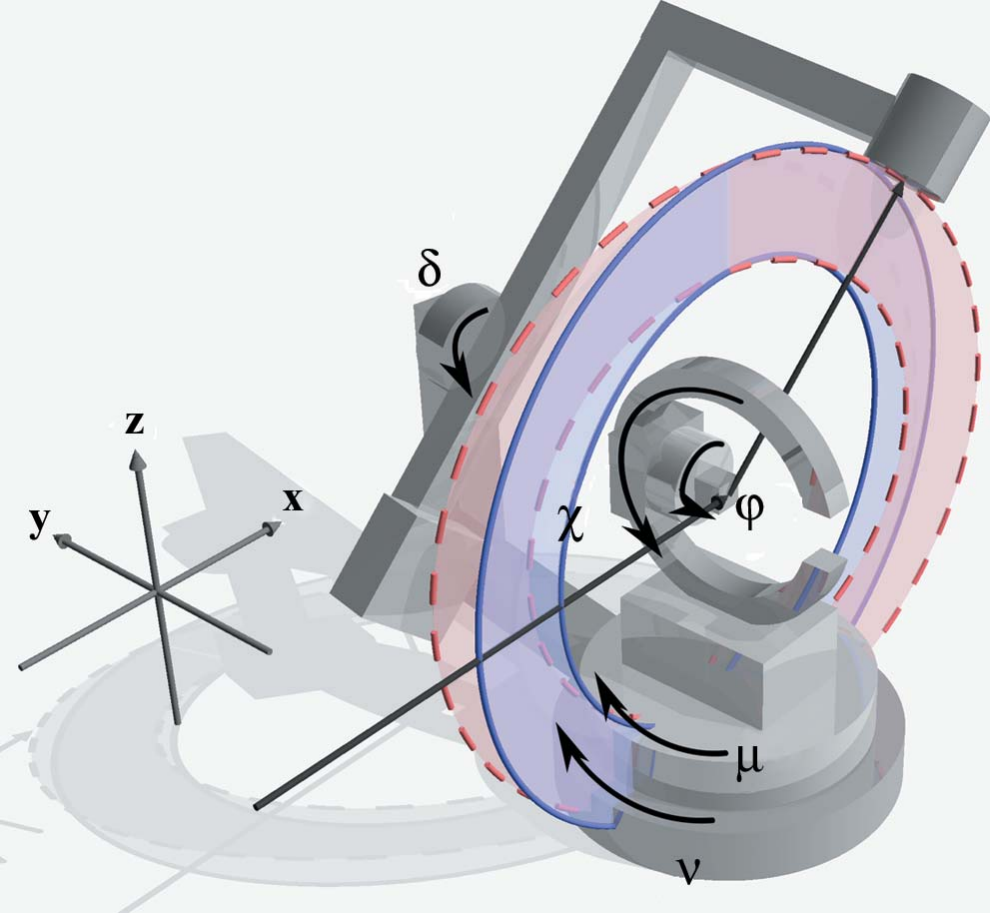
Sketch of a goniometer with three sample axes (μ, χ, ϕ) and two detector rotations (ν, δ). The red and blue planes circumscribed by a dashed and a solid line, respectively, indicate the detector rotation planes of the inner detector rotation for two different positions of the outer detector arm rotation.

To elucidate the definition of a goniometer we use the goniometer shown in Fig. 2 ▶. The goniometer has a 3S + 2D geometry, which means it offers three degrees of rotation for the sample and two independent degrees of freedom for the detector. The goniometer axes are specified by their axis direction and rotation sense. The coordinate system is chosen to have **x** pointing downstream along the primary beam, **z** is pointing upwards and **y** is pointing backwards to have a right-handed reference frame. In *xrayutilities* we describe each rotation axis with one character giving the axis direction (either 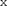, 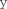 or 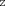) and another the rotation sense (either 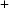 or 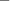). This description needs to be supplied for the sample and detector circles for the case where all axes are at zero position starting with the outermost circle. The goniometer in Fig. 2 ▶ has three sample circles (μ, χ, ϕ) with the indicated rotation directions. The outermost angle μ turns clockwise around the *z* axis and thus is described by ‘
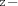
’. The complete sample goniometer is described by the following set of rotations: (‘
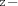
’, ‘
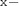
’, ‘
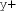
’). For the detector circles turning around the *z*- and *y*-axis directions, the corresponding definition is (‘
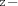
’, ‘
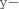
’). A full example of how to insert these definitions into *xrayutilities* is given in Appendix *B* [App appb].

### 2.2. Special rotation directions (kappa goniometer)

In addition to rotations around axes of the coordinate system, an often used goniometer geometry is the so-called κ geometry (Poot, 1972 ▶; Thorkildsen *et al.*, 1999 ▶), in which one of the rotation axes has an angle of typically 45–60° with one of the other axes. In *xrayutilities* we define such an axis by 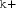 or 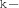. The plane of the κ rotation axis and its angle with respect to a reference direction are specified in a configuration file by the options 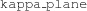 and 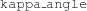. The rotation matrix needed in matrix 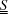 can be determined easily using the general equation given in Appendix *C* [App appc].

## 3. Angular to momentum space conversion for one- and two-dimensional detectors

Equation (5) describes the conversion from angular coordinates of a general goniometer to reciprocal space for a point detector only. The generalization for a linear or area detector requires information about the pixel size and distance, and the direction into which a pixel row extends. Usually in the data files only one angular position is stored for every data point recorded with a multidimensional detector. This angular coordinate corresponds to the position of the so-called center channel/pixel (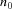), which is the pixel hit by the primary beam when all the angles are set to zero. For all other detector pixels their position needs to be determined. This is easiest for the case of a curved one-dimensional detector in which every detector channel or pixel covers the same detector angle segment. Every detector pixel (identified by the channel number *n*) corresponds to a detector angle 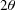 given by
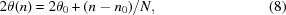
where *N* is the number of channels per unit of angle of the detector circle and 2θ_0_ the detector angle of the center channel *n*_0_. When the detector angle is expressed in degrees, *N* equals the number of channels per degree of rotation.

**Figure 3:**
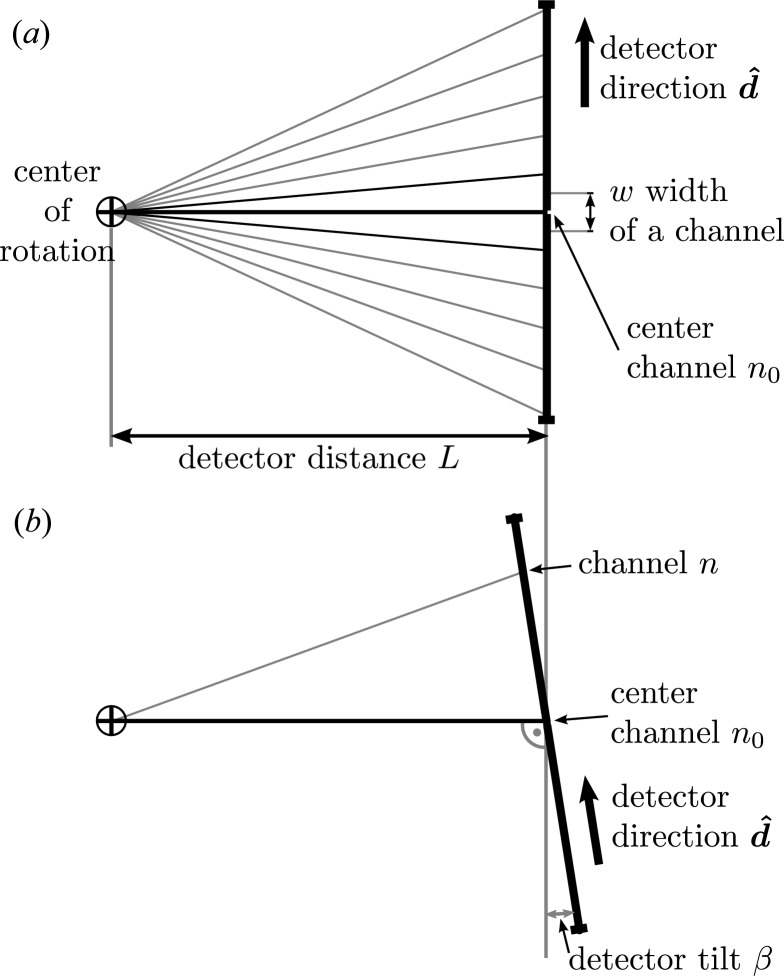
Sketch of a linear detector mounted at distance *L* from the center of rotation of the goniometer. In (*a*) the detector direction specifying the direction along which higher channel numbers are found is given by 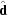. Also indicated is the width of one channel *w* and the center channel 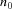, which is the channel where the primary beam is centered at zero detector angle. Part (*b*) shows the possible detector tilt β (misalignment) resulting if a detector is not mounted perfectly perpendicular to the X-ray beam.

Most modern detectors are, however, straight as shown in Fig. 3 ▶ and not curved, and therefore equation (8) is not generally applicable. It is only in the limit of a large sample–detector distance that the curved and straight detectors become indistinguishable. Nevertheless the channel per degree approximation is frequently used in practice. In *xrayutilities* one-dimensional detectors are treated as straight detectors and equation (5) is adjusted accordingly. For each detector pixel *n*, the corresponding direction of a scattered beam hitting this pixel is calculated, replacing equation (4) by
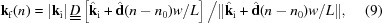
where *w* and *L* are the size of a detector pixel and the distance from sample to detector, as shown in Fig. 3 ▶. The direction in which the detector channel number increases is given by 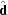. A ‘hat’ on a vector indicates a unit vector. The fraction 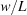 is in the case of large sample to detector distance equal to 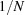, where *N* is the number of channels per radian, and equation (9) effectively simplifies to the form of equation (8). For a two-dimensional detector with channel directions 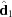 and 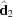 we can write an equivalent equation for the exiting wavevector of channel (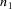, 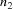) including the width of the detector pixels 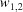 as 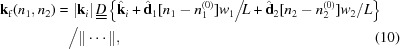



Using the description of the detector in real space we therefore avoid the ‘channel per degree’ approximation, which implicitly assumes that a detector is curved and therefore does not work for small sample–detector distances with large detectors. Inserting equations (9) and (10) into equations (2) and (3) yields general equations for the reciprocal space conversion of linear and area detectors. The possible mis­align­ments are included in those equations *via* the pixel directions 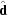 for the one-dimensional and 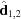 for the two-dimensional detectors.

### 3.1. Detector parameters of one-dimensional detectors

**Figure 4:**
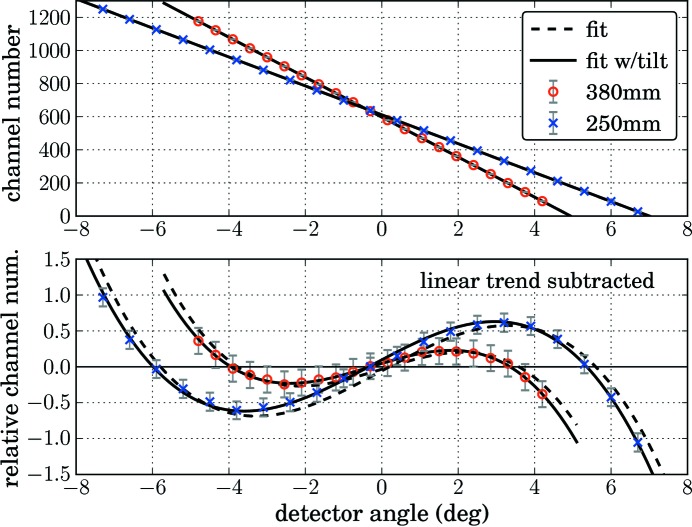
Determination of the ratio 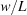 and center channel for a linear detector with pixel size of 50 µm from the variation of the beam position during a scan through the primary beam. The upper plot shows the channel numbers at which the primary beam is observed during the scan *versus* the detector angle. Furthermore the fits of equation (12) to the recorded data are shown by black lines. The lower plot shows the same data set and fits but with the linear trend subtracted. A fit with (full line) and without (dashed line) considering a detector tilt is shown for two detector distances of 250 and 380 mm. In the upper plot these two fits are indistinguishable.

For the conversion algorithms described above several detector parameters are needed, among them the detector distance *L* and width of one detector channel *w*. Since neither the detector distance nor the width of one channel can be easily measured very accurately, we use the fact that only their ratio is needed, which can be determined from an angular scan through the primary beam. Assume a linear detector is mounted along the direction in which the detector arm of the used instrument moves. Scanning the detector angle will move the primary beam over the detector, and by modeling this movement we are able to determine the needed quantities. Therefore assume a linear detector mounted at a distance *L* like the one shown in Fig. 3 ▶. Neglecting for the moment a possible detector tilt, *i.e.* when a detector is not mounted perfectly perpendicular to the X-ray beam, the detector channel at which the detector is hit for a detector arm angle 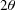 is given by 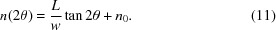
If a detector tilt β as shown in Fig. 3 ▶ is included, the above equation needs to be modified, and one finds
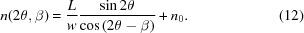
By fitting one of the two models one can find the detector parameters needed for the reciprocal space conversion of a linear detector. For this purpose, a scan through the primary beam should be performed with the linear detector, and the detector spectra should be saved at every position. Since not only the slope is determined (which only needs two spectra), it is necessary to acquire several spectra: we suggest typically 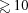. For the determination of the parameters two functions are provided in *xrayutilities*. One of them automatically processes the spectra of a linear detector acquired during a scan through the primary beam and determines the position of the primary beam in every spectrum by fitting a Gaussian peak. From this fitting the position of the primary beam is found with sub-pixel precision. The second function needs the user to determine the channel number of the primary beam and supply it to the function, which should be used only in cases where the first function is not applicable. Calling one of the two functions will produce a plot like the one shown in Fig. 4 ▶, which shows the channel number at which the beam hit the detector together with the variation expected from the model(s). A second plot shows the comparison of model and measured data with the linear trend subtracted, for two different detector distances of 380 and 250 mm for a straight linear detector with a pixel size of 50 µm. The different distance shows up as a different slope in the upper plot. When the linear trend is subtracted the nonlinearity due to the trigonometric functions in equations (11) and (12) can be seen. The fit is also sensitive to a tilt of the detector from the direction perpendicular to the primary beam, as can be seen from the comparison of the model without tilt [equation (11), dashed lines] and the model with tilt [equation (12), full lines]. For the measurements shown in Fig. 4 ▶ an artificial tilt of 0.3° was introduced, which was also determined by the fit. Note that a tilt of the detector can result not only from a not perfectly mounted detector but also from the fact that experimentalists choose to use a center channel that does not correspond to the true center of the detector and do this by redefining the zero position of the detector arm rotation, which introduces a tilting of the detector by exactly the angle by which the detector arm angle changed. Using a translation of the detector perpendicular to the beam (mounted on top of the detector arm) would prevent such a tilt; this option is, however, very often not available.

**Figure 5:**
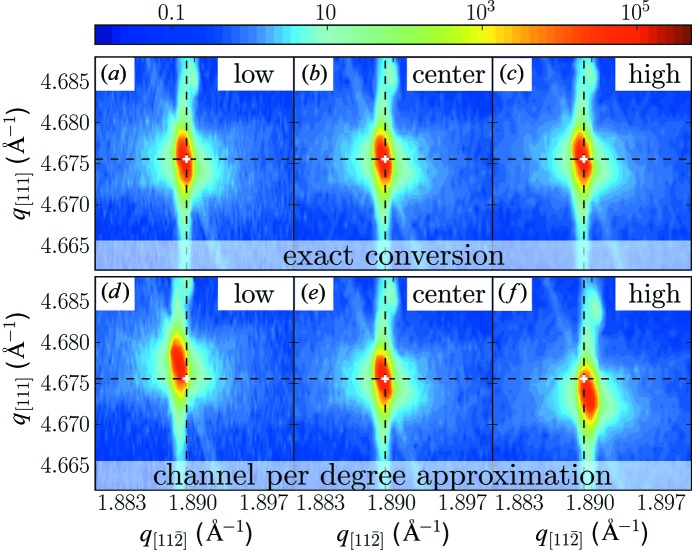
Three reciprocal space maps recorded around the Si (331) Bragg peak using the same sample movement but using three different parts of a linear detector for detection and two different descriptions of the linear detector for reciprocal space conversion. The white cross marks the nominal position of the Si (331) Bragg peak. Panels (*a*)–(*c*) were converted using the exact reciprocal space conversion described in the text, whereas for panels (*d*)–(*f*) the same measurements were converted using the ‘channel per degree’ approximation, leading to errors in the observed peak positions. The measurements shown in panels (*a*), (*d*) were recorded when the detector was offset to lower angles, and those in panels (*c*), (*f*) with an offset to higher angles, whereas the reciprocal space maps in panels (*b*), (*e*) were recorded with the signal centered on the detector.

Basically the discussion so far shows the necessity of nonlinear models instead of the simpler linear fitting, which is a reasonable approximation only in the case of a large sample to detector distance. If a linear or area detector covers an angular range of 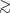
8° it becomes absolutely necessary to use the above treatment since deviations would exceed one channel for typical channel sizes of around 50 µm. Using large linear detectors at moderate sample–detector distances this limit is frequently reached, especially in modern laboratory diffractometers. To further highlight the necessity of the nonlinear models we show an example of an X-ray diffraction reciprocal space map measurement of the Si (331) Bragg peak of an Si(111)-oriented single crystal in Fig. 5 ▶. The measurement was performed with the above-mentioned linear detector at a distance of 250 mm using a laboratory diffractometer with Cu *K*α_1_ radiation produced by a Ge(220) channel-cut monochromator. The same measurement was repeated three times; however, different parts of the detector were used for the detection of the diffracted signal. We compare the reciprocal space maps obtained using our exact conversion formalism with the ‘channel per degree’ approximation, which assumes a curved detector as described by equation (8). Using this approximation (Figs. 5 ▶ *d*–5 ▶ *f*) we find that only the measurement using the central part of the detector gives the correct peak position in reciprocal space/lattice parameter. The measurements performed with the detector offset to higher or lower angles would result in a lattice parameter wrong by approximately 0.04%; this is far beyond the usual sensitivity of the experimental setup, which is said to be <1 × 10^−4^ and can be increased further, as for example outlined by Fewster (1999 ▶). Using the accurate conversion no such shifts are observed and the three measurements (Figs. 5 ▶ *a*–5 ▶ *c*) are indeed equivalent.

### 3.2. Detector parameters of two-dimensional detectors

For two-dimensional detectors a similar determination of the 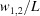 ratio can be performed if the detector rotation and other misalignments (see below) are negligible, by decomposing the problem into two separate one-dimensional problems. In the case where the detector is rotated around the primary beam the problem can no longer be decomposed. In particular one more problem specific to two-dimensional detectors arises. Very often the true zero of the outer detector arm rotation is not known. For the inner detector rotation this does not imply any particular problem since it shows up only as additional tilt, which can be easily corrected. However, an offset in the outer detector rotation implies a rotation of the rotation axis of the inner detector rotation. If the outer motor is not at the true zero the inner rotation will no longer rotate perpendicular to the primary beam, as indicated by the two rotation planes shown in Fig. 2 ▶. If the outer motor were at its true zero the detector would rotate in the blue plane; owing to an offset in the outer rotation the detector instead rotates in the red plane. This means that the offset of the outer motor needs to be determined from alignment scans as well.

**Figure 6:**
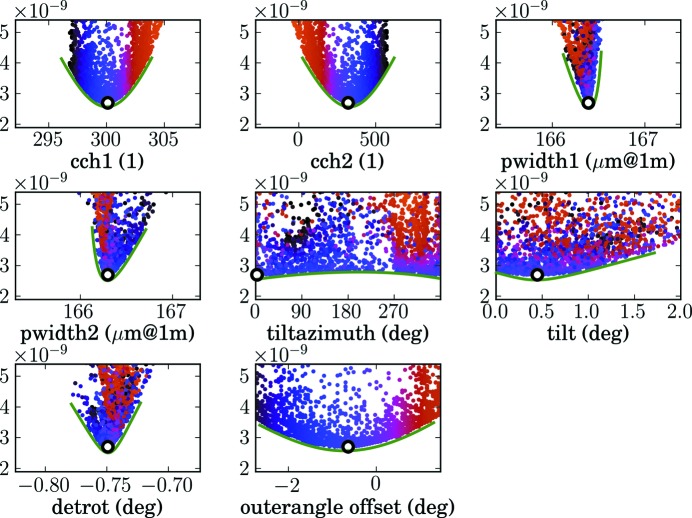
Determination of detector parameters. Shown is the optimization error *versus* the eight considered parameters: the center channels of the detector and the width of the pixels in both directions, the detector tilt (azimuth and tilt angle), the detector rotation, and the offset of the outer goniometer stage. The found optimum is marked by a black circle. All other points correspond to fits that did not reach the global minimum; these were produced from various different starting parameters of the fit. Green lines are guides to the eye to visualize the minimum found in all the parameters. The points of the fits are colored to enable the identification of correlations.

In general one needs to determine eight parameters for a two-dimensional detector: the center channels [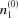, 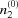], the ratios 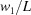 and 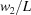, and the directions of the vectors 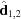, which are specified by the tilt, tilt direction and rotation of the detector around the primary beam and the outer angle offset described above. The untilted directions of the vectors 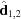 can be determined unambiguously by knowledge of the primary beam and detector rotation directions and therefore do not need to be included in the fit. Similarly as for the one-dimensional detector, these parameters can be determined from scans through the primary beam. It is necessary to use at least two scans, one with the inner and one with outer detector arm rotation with sufficient points around the primary beam, and to acquire a detector image at several positions. From the primary beam position observed in those images the detector directions and other necessary parameters can be determined by a fitting routine. As quality criterion of the fit the average 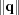 value of the pixel at which the primary beam is observed is used. This position is calculated using equations (3) and (10). Since we observe the primary beam in all the images, this 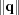 value should be zero when the correct detector parameters are found. *xrayutilities* provides a function that takes the detector images and performs a fit of the eight described parameters. Since several of these parameters, *e.g.* the offset of the outer motor with one of the center channels, are coupled with each other, the fit is performed in such a way that it starts from several starting parameters to find the global minimum in the parameter space. An example of such a fit is shown in Fig. 6 ▶. The detector parameters have been determined from two perpendicular scans through the primary beam using a Maxipix detector with effectively 516 × 516 pixels at beamline ID01, ESRF, Grenoble, France (ESRF, 2013*a* ▶). One scan was performed using the outer detector arm motor (moving horizontally) and the other one with the inner detector circle (moving vertically). In each scan we used 70 points, which means that in total 140 images were used. Note that using considerably fewer points does reduce the quality of the fit until no reliable statement can be made about the misalignment parameters. It is therefore suggested to use a comparable number of points to that used in this example. Furthermore the images were manually selected to use just those with the full primary beam in the active area of the detector. These images were fed into the algorithm described above, and the detector rotation, tilt, tilt azimuth and outer angle offset along with the center channels and detector pixel size were determined. This determination is shown in Fig. 6 ▶, where the average 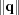 deviation of the detector pixel positions associated with the primary beam in the performed scans is shown. The value of this deviation is approximately the offset of the absolute value of the scattering vector in reciprocal space. This deviation shows a clear minimum in all eight parameters. These eight parameters are as follows:

(1, 2) cch1,2 are the center channels of the detector in both directions. This is the pixel number where the primary beam is hitting the detector at the true zero position of the detector arm (including the outer angle offset).

(3, 4) pwidth1,2 are the pixel widths of the pixels in the two detector directions. The unit of these values in the plot is the size of the pixels in micrometres, assuming a detector distance of 1 m. This corresponds to the parameter 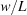 from above. If the pixel size is known, the detector distance can be calculated, or *vice versa*.

(5) tiltazimuth is an angle giving the direction of the detector tilt. Values of 90 and 270° correspond to a tilt rotation around the first pixel direction and 0 and 180° to rotation around the second pixel direction.

(6) tilt is the tilt angle of the detector plane around the direction given by tiltazimuth; since tiltazimuth runs from 0 to 360°, only positive tilts are used.

(7) detrot is the detector rotation around the primary beam direction in degrees.

(8) outerangle offset is the offset of the outer detector arm rotation in degrees.

For the determination of these parameters, fits with various different starting parameters are performed. This is absolutely mandatory since several of the parameters are correlated, and therefore a single fit would not necessarily find the global optimum. One correlation that can be easily imagined is the correlation of one of the center channels with the outer rotation offset. Since the detector orientation is not given to the script and is automatically determined from the given scan data, it is not *a priori* clear if this is center channel 1 or 2. In Fig. 6 ▶ this correlation can be seen in the plot showing center channel 2 (cch2) and the outer angle offset. The cloud of points from center channel 2 is rather broad since this value changes when the outer angle offset is changed. In fact the coloring of the cloud of points of center channel 2 reveals that it is only mirrored with respect to the data of the outer angle offset. A not so intuitive correlation is that of the detector tilt with the outer angle offset. Imagine a not tilted detector, which is however offset in the outer angle. Such an offset will effectively tilt the detector by exactly the offset in the outer angle with a tilt azimuth of 90 or 270°. If the detector is already slightly tilted in an arbitrary direction without outer angle offset, the tilt due to an outer angle offset will overlay with the initial tilt and influence the tilt azimuth and tilt in a nontrivial manner. The optimal set of parameters for the area detector of beamline ID01 was determined from the point with the lowest error (global minimum in the parameter space) of 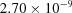.

To elucidate the benefit of considering these ‘misalignment’ parameters, which are usually omitted, we also give the obtained errors when several of the quantities are fixed. When only the center channels and pixel widths are fitted we obtain an error of 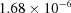, which is orders of magnitude higher than our optimal error. In the present example the most important parameter is the detector rotation, which brings the error down to 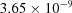. The second most important parameter is the outer angle offset, which brings down the error further to 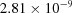. If only the tilt (tilt azimuth and tilt angle) or the outer angle offset are fitted without the detector rotation, the error can only be reduced by less than 10% from the value without considering any misalignment. Only when all parameters are considered in the fit can the error be reduced to the optimum. This clearly shows that all these parameters should be considered to obtain the correct reciprocal space positions of the full area detector.

It should be noted that some of the parameters like the offset of the outer detector arm rotation and detector tilt can only be determined when the detector is spanning a certain angular range; the user is therefore urged to check the resulting plots in order to see if the parameters are reasonable. In cases where the detector distance is large the outer angle offset can only be determined with large error, and it might be better to fix the corresponding parameter during fitting. In cases when the detector tilt is small the tilt azimuth will not have a clear minimum and is therefore an indeterminable parameter. The script also outputs the code needed for initialization of the area detector, which considers the determined tilts and rotations as shown in Appendix *B* [App appb]. These detector tilts of linear and area detectors are then included in the reciprocal space conversion. When the detector pixel position is calculated as described in equations (9) and (10), the detector direction 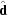 or 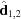 needs to be rotated accordingly before the detector position is calculated.

If no detector rotations are available, *e.g.* when using an area detector for powder diffraction, we refer to the *Fit2D* software (Hammersley, 2013 ▶) for determining the necessary parameters.

Note added in proof. The sensitivity of our method with respect to the outer angle offset can be increased significantly when, in addition to scans in the primary beam, also the movement of a known Bragg peak of a reference crystal is analyzed. We have added a function enabling this, and refer the reader to the documentation of our package for more details.

## 4. Conclusions

In conclusion, we present algorithms for reciprocal space conversion of X-ray diffraction data. We generalize the equations given by Busing & Levy (1967 ▶) and You (1999 ▶) for the use of linear and area detectors. Using our approach we can convert angles from arbitrary goniometers to reciprocal space coordinates. For the conversion of linear and area detectors several detector parameters, including all possible misalignments, are needed. Recipes were presented allowing these parameters to be determined from alignment scans for both linear and area detectors. The software package including the presented algorithms is available at http://xrayutilities.sourceforge.net. The algorithms were shown to determine detector parameters of one- and two-dimensional detectors that are otherwise not determinable.

## Appendix A Implementation details

The software package is available from http://xrayutilities.sourceforge.net and is mainly coded in the popular scripting language Python with some performance-critical parts written internally in the C programming language. The user needs to use only the Python package. It is worth noting that Python has recently gained a lot of attention and that several other diffraction-oriented packages have also been written in Python and published as open-source software (Sørensen *et al.*, 2012 ▶; Kieffer & Karkoulis, 2013 ▶; Juhás *et al.*, 2013 ▶; Micha, 2013 ▶; ESRF, 2013*b* ▶). Installation of *xrayutilities* is possible on all major platforms (Windows, Mac, Linux/Unix). In addition to the reciprocal space conversion the package offers several functions to read data from *spec* files, from *spectra* files, and from laboratory diffractometers of Panalytical (xrdml) and Seifert (nja) as well as several CCD data files (edf, tiff) and other functions, which are described in the documentation found at the web site.

The reciprocal space conversion’s performance-critical part, which implements equation (5) and equivalents for linear and area detectors, is coded in C. As can be seen from equation (5), this part mainly consists of  matrix operations. For the conversion of angular positions the matrices of the sample rotations need to be set up, for which rotation matrices like the one shown in equation (7) are used. These matrices are multiplied in the order given in equation (6). Furthermore, the conversion involves one matrix inversion, which is performed using the adjugate matrix formula. For matrix  this means the inverse is given by

Depending on the number of samples () and detector circles () to be considered in the conversion for a point detector,  matrix multiplications, the matrix inversion and one matrix–vector multiplication as well as one matrix–matrix subtraction need to be performed for every data point.

However, for linear and area detectors the complete conversion does not need to be done for every detector pixel. For one spectrum or image all the sample and detector angles are considered to be constant and therefore the  matrix multiplications as well as the matrix inversion need to be performed only once. Only two matrix–vector multiplications and some vector arithmetic to set up the pixel position need to be performed for every detector channel/pixel, which enables a fast conversion even for large area detectors. Furthermore, the conversion for multiple data points is independent and can therefore be easily performed in parallel on modern multiprocessor computers.

## Appendix B Example script for the reciprocal space conversion

In the following an example script for the conversion of an image from an area detector recorded using the goniometer shown in Fig. 2 ▶ is given. This goniometer has the sample angles μ, χ and ϕ and detector angles ν and δ. The rotation axes of this goniometer are as given in the main text [sample circles: (‘’, ‘’, ‘’); detector circles: (‘’, ‘’)] and the primary beam propagates along the positive *x*-axis direction. The script is written in the Python script language.

All the detector parameters (such as center channels, number of pixels, size of the pixels and the distance as well as possible tilts and rotations of the detector) need to be given to the  function. These parameters are the result of a fit like the one shown in Fig. 6 ▶. The conversion is then performed by calling the  method of an experiment class object, where also the outer angle offset needs to be considered. An experiment object holds information about the experiment, such as the orientation of the crystal on top of the innermost sample circle, the goniometer geometry and the X-ray energy. Here the object is initialized for an experiment performed using X-rays with an energy of 9000 eV and a silicon crystal with the [100] direction along the primary beam at zero position and [010] along the *z* axis. The second argument [010] gives the reference direction perpendicular to the primary beam in the plane spanned by the innermost detector direction. Dummy values for the reference directions can be used when the  function is not used.

After execution of this script the variables ,  and  hold the reciprocal space position of the detector pixels specified by the region of interest variable. In the case above these variables will have a shape of 400 × 400, holding the *q* position of the pixels 100–500 in both detector directions. The variable  could be used to reduce the number of data by block averaging. Using  effectively quadruples the area covered by one pixel and therefore reduces the number of returned *q* positions to 200 × 200 per coordinate.

Furthermore the use of the algorithms to directly convert angles to reciprocal space indices *h*, *k* and *l* is shown. A function named  is called together with the orientation matrix to convert the experimental angles directly to the indices *h*, *k*, *l* instead of the scattering vector in units of Å^−1^. Instead of specifying the orientation matrix one can also use the crystallographic orientation of the sample to determine the matrix , which is the default if no  matrix is explicitly given. Special algorithms also exist for determining the matrix  from unit-cell vectors. In the example, the matrix  is determined directly from the built-in material properties of silicon. In the present case (horizontal diffraction) the scattering vector for the area detector has mainly a component along the negative *y*-axis direction, whereas the *h*, *k*, *l* components have their largest components along *l*.

Experimental angles, of course, do not need to be entered manually. They can be read from several different data formats as mentioned above. Details are given in the documentation found online.

## Appendix C Rotation matrix for arbitrary axis direction

Several rotations need to be performed around non-primitive directions, *e.g.* in the case of κ goniometers or when calculating detector tilts. In such cases the rotation axis and the angle of rotation are known, and rotation matrices are generated using a formula given by Lang & Pucker (1998 ▶). The components *r_ij_* of the rotation matrix  for a rotation of angle α around the axis  are given by

with the Kronecker delta  and the Levi–Civita symbol .
